# Neoadjuvant treatment with lenvatinib and pembrolizumab in a *BRAF* V600E-mutated anaplastic thyroid cancer: a case report

**DOI:** 10.3389/fendo.2024.1389294

**Published:** 2024-07-09

**Authors:** Daniele Barbaro, Raffaella Forleo, Maria Antonietta Profilo, Paola Lapi, Carlotta Giani, Liborio Torregrossa, Elisabetta Macerola, Gabriele Materazzi

**Affiliations:** ^1^ Endocrinology ASL Nord-West, Spedali Riuniti, Livorno, Italy; ^2^ Department of Surgery, UO Pathology, University of Pisa, Pisa, Italy; ^3^ Department of Surgery, Endocrine Surgery, University of Pisa, Pisa, Italy

**Keywords:** anaplastic thyroid cancer, lenvatinib, pembrolizumab, *BRAF* mutation V600, neoadjuvant

## Abstract

**Background:**

Tyrosine kinase inhibitors (TKIs) and immunotherapy have been proposed for advanced metastatic anaplastic thyroid cancer (ATC). We report a case of *BRAF* V600E-mutated ATC in which lenvatinib (L) plus pembrolizumab (P) enabled neoadjuvant treatment.

**Case presentation:**

A male patient aged 65 years presented with a rapidly enlarging left latero-cervical mass. Fine needle aspiration was suggestive of ATC. Surgical consultation excluded radical surgery. While awaiting molecular profile analysis and considering the fast evolution of the disease, treatment with L and P was started. L was started at a dose of 14 mg daily, while P was started at the standard regimen (200 mg every 3 weeks). After 1 month, computerized tomography showed a reduction in the mass with almost complete colliquative degeneration, and the carotid artery wall was free from infiltration. Radical surgery was performed. Histology confirmed papillary thyroid cancer (PTC) in the left lobe and ATC with extensive necrosis in the left latero-cervical lymph node metastasis. The margins were free of tumors (R0). A *BRAF* V600E mutation was present in both PTC and ATC. At the 1-year follow-up, the patient was free of disease.

**Conclusion:**

L and P in combination also appeared to be effective as a neoadjuvant treatment for *BRAF* V600E-mutated ATC. This combination treatment could be used when there is an opportunity for complete resection of the cancer, and as soon as possible. The intermediate dose of 14 mg of L appeared to be well tolerated and effective.

## Introduction

Anaplastic thyroid cancer (ATC) is one of the most aggressive human cancers. It usually presents at an advanced stage, and the median survival is approximately 4 months (1, 2). Complete resection can be achieved in only a minority of patients. Incomplete resection followed by multimodal therapy, i.e., external beam radiotherapy (EBRT) and chemotherapy or EBRT alone, has been shown to ameliorate the prognosis in some but not all studies, above all depending on the disease stage (1–3). Recently, tyrosine kinase inhibitors (TKIs) and immunotherapy have been proposed and used for advanced metastatic ATC (1, 2, 4–9). A number of interesting reports have shown that the combination of TKIs and pembrolizumab (P) can have an impact on survival in advanced and metastatic forms. The use of highly selective TKIs is dependent on the presence of specific mutations in the tumor. Dabrafenib (D) and trametinib (T) especially, block selectively and consequently two enzymes of the MAP kinase cascade, and they have been used in *BRAF* V600E-mutated ATC. The use of D and T has significantly changed the treatment of ATC (10); in fact, the molecular profile is one of the most important first diagnostic tools in these cancers. On the other hand, the multi-TKI lenvatinib (L) has been used in *BRAF* wild-type cases. In almost all cases published, TKIs plus immunotherapy have been used in metastatic cancer (stage IVC) after surgical intervention and/or after EBRT and chemotherapy. In a few cases, this association was used for neoadjuvant purposes, but no patients were cured after 1 year. We report a case of ATC in which L+P enabled neoadjuvant treatment followed by a complete resection of the tumor, with the patient free of disease at 1 year. In this case, L was also used, although the cancer was *BRAF* V600E-mutated.

## Case report: diagnosis, treatment, and follow-up

**Figure 1:**
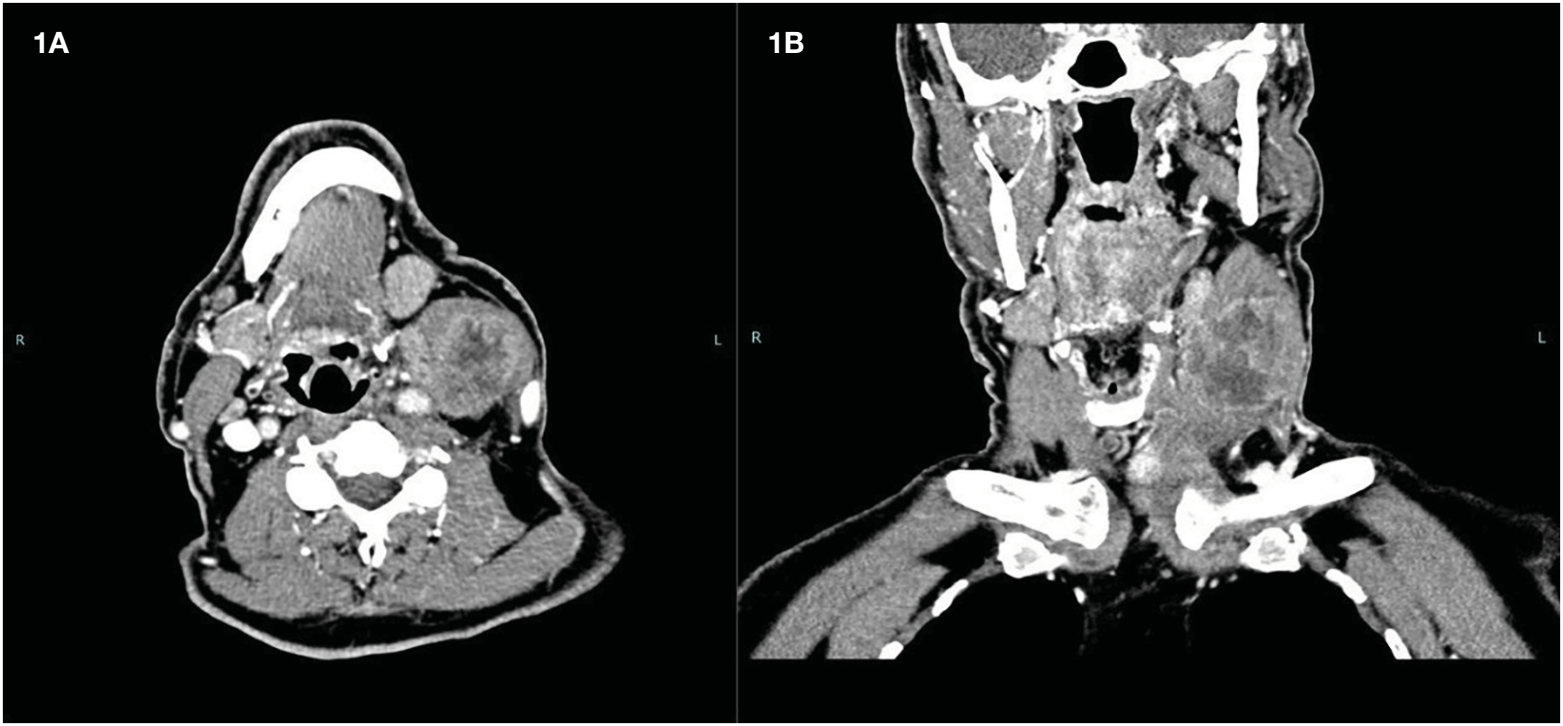
Latero-cervical mass before treatment. **(A)** Transverse scan. **(B)** Coronal scan.

A male patient, aged 65 years, presented with a rapidly enlarging left latero-cervical mass. The mass was hard/wooden, fixed to the neck structure, and extended from the jaw angle to the base of the neck. Ultrasonography showed a hypoechoic mass with a maximum size of approximately 7 cm in the latero-cervical II and III compartments and a hypoechoic nodule with a maximum size of 2 cm in the left thyroid lobe. Fine needle aspiration (FNA) showed a smear suggesting papillary thyroid cancer (PTC) on the thyroid nodule and ATC on the latero-cervical mass (confirmed by a large needle biopsy). CT showed a large mass with a colliquative component (**Figure 1**), completely infiltrating the jugular vein and, most importantly, infiltrating the carotid artery wall. A double surgery consultation excluded the possibility of radical surgery and raised some doubts regarding the outcome of a surgical intervention.

**Figure 2:**
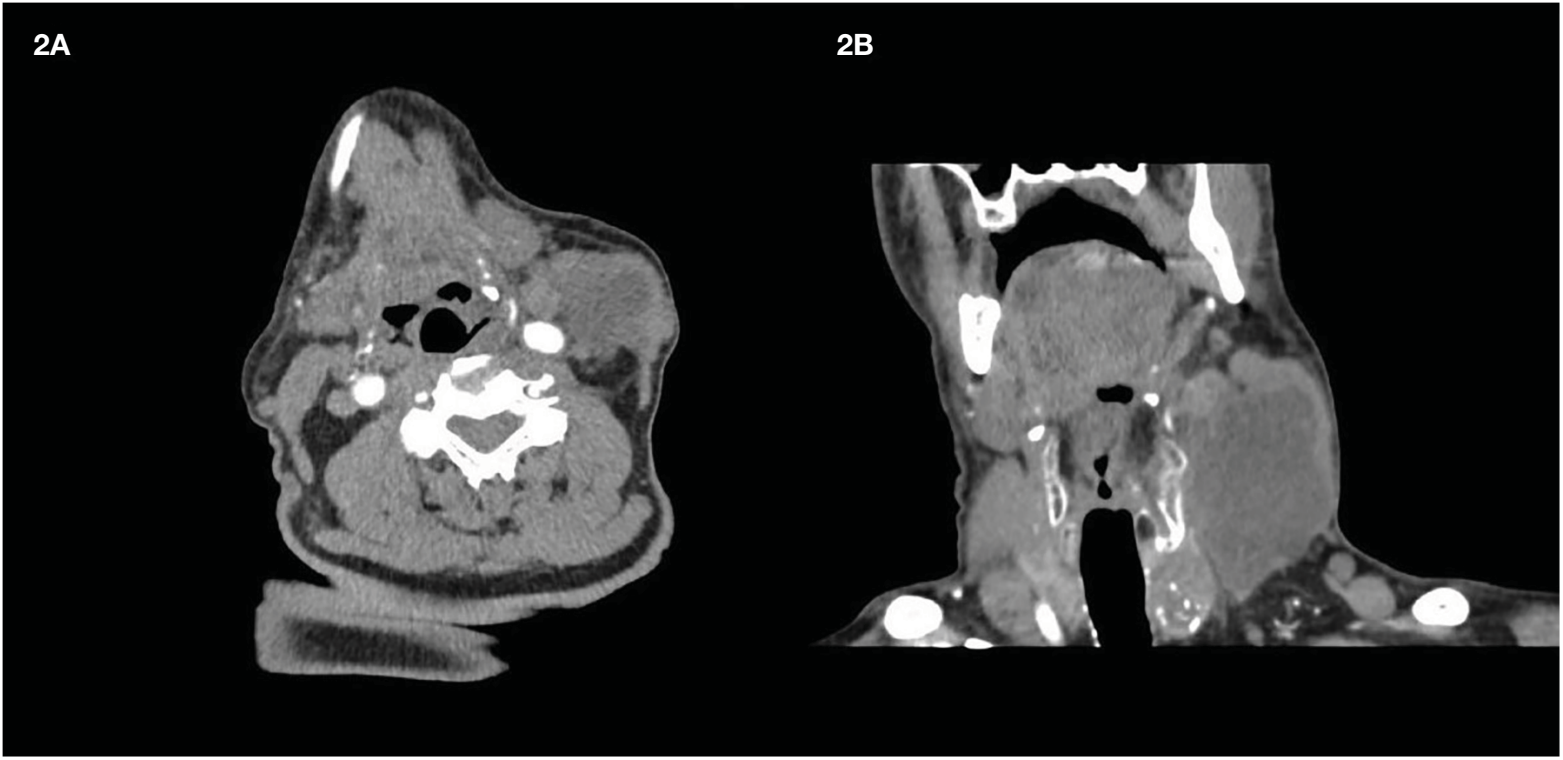
Latero-cervical mass after 1 month of treatment. **(A)** Transversal scan. **(B)** Coronal scan. The transverse scan shows the detachment from the carotid artery wall. Almost the whole mass appears dark due to the presence of colliquative necrosis.

While awaiting the results of the molecular profile analysis performed on the smear and considering the fast evolution of the disease, treatment with L and P was started. Based on our previous experience (11), L was started at a lower dose, i.e., 14 mg instead of 24 mg, and P was started at the standard regimen (200 mg every 3 weeks). After 1 month (specifically 32 days after starting L and 10 days after the second infusion of P), clear clinical benefits were observed: a slight visual reduction and, most importantly, a change in the consistency of the mass, which now appeared soft and mobile. CT showed a mild reduction, but with almost complete colliquative degeneration and with the carotid artery wall free of infiltration (**Figure 2**).

Surgical resection was then performed 8 days after the discontinuation of L and when complete macroscopic resection was possible. The crucial issue after the surgical intervention was whether or not to continue with the treatment. While also awaiting the results of histology, it was decided to continue with L+P; thus, therapy was restarted after 8 days. However, possibly due to the large extent of the surgical intervention (removal of the sternocleidomastoid muscle and the jugular vein), wound dehiscence was observed after 2 weeks, and all drug treatments had to be stopped.

Histology confirmed the coexistence of differentiated and undifferentiated tumor histotypes. In the right lobe, three PTC microfoci (maximum size, 0.2 cm) were detected, along with two lymph nodes with PTC metastases. In the left lobe, a 3-cm classic PTC and a 2.4-cm tall cell subtype PTC were observed. Three perithyroidal lymph nodes were positive for PTC metastases, while the supraclavicular lymph nodes sampled were free from metastases. The residual tumor mass infiltrating the pre-thyroidal muscles was reported as ATC with extensive tumor necrosis. On the other hand, in a large left latero-cervical lymph node metastasis, the coexistence of differentiated and undifferentiated tumor components was observed. The excision margins were free of tumor (R0). Considering the R0 resection, medical treatment was not started again after the recovery of the wound dehiscence. To investigate the relationship between the differentiated and undifferentiated tumors, molecular analysis of the *BRAF*, *TERT*, and *TP53* genes was performed separately on the two tumor components of the latero-cervical lymph node metastasis. *BRAF* V600E and *TERT* promoter mutations were present both in differentiated thyroid cancer and in ATC, while the *TP53* mutation was detected only in ATC. Details on the genetic profile of the tumors are reported in **Table 1**.

**Table 1:** Molecular profile of the tumor.

Marker	PTC	ATC	Methodology
*BRAF*	POSITIVE V600E mutation	POSITIVE V600E mutation	NGS on FNA; real-time PCR to confirm the results on tissue samples
*TERT* promoter	POSITIVE C250T mutation	POSITIVE C250T mutation	Direct sequencing
*TP53*	NEGATIVE	POSITIVE M246I mutation (exon 7)	Direct sequencing
PD-L1	Not performed	POSITIVE 80% (TPS)	Immunohistochemistry (SP263 clone)

Molecular analysis was conducted separately on the differentiated (PTC) and undifferentiated (ATC) tumor components.

PTC, papillary thyroid carcinoma; ATC, anaplastic thyroid carcinoma; NGS, next-generation sequencing; FNA, fine needle aspiration; TPS, tumor proportion score.

**Figure 3:**
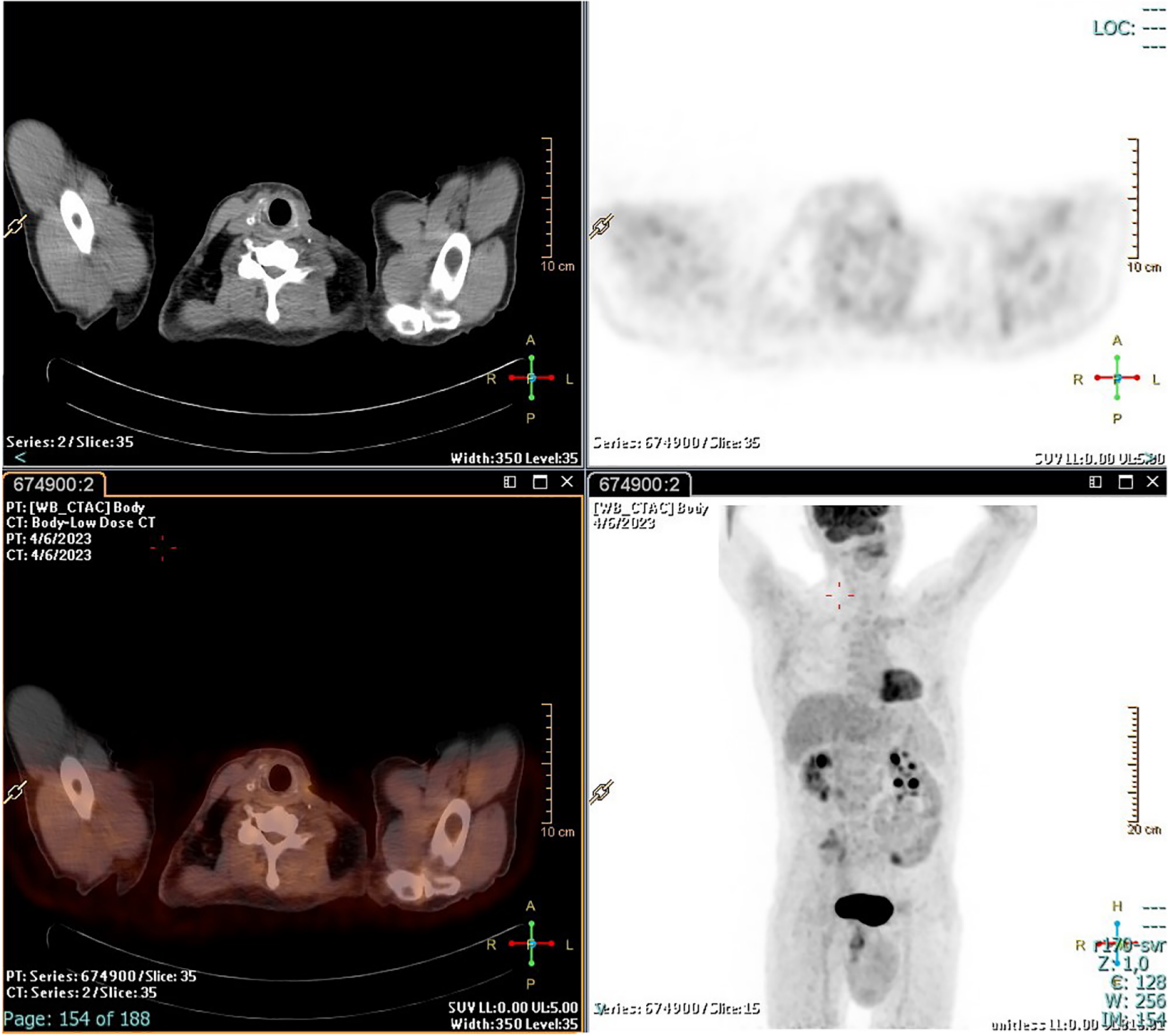
^18^FDG/PET-CT scan after 4 months. No pathological uptake.

**Figure 4:**
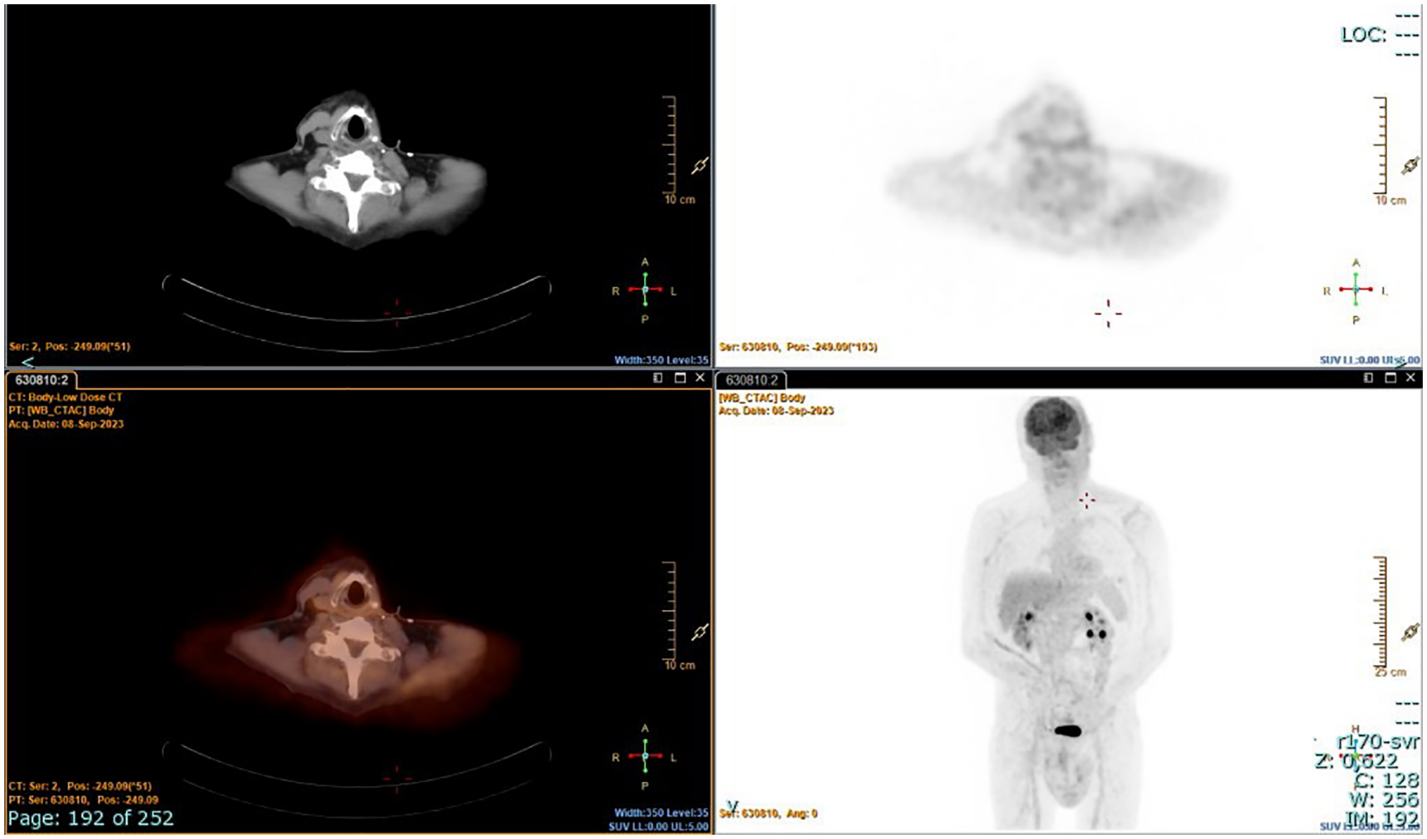
^18^FDG/PET-CT scan after 12 months. No pathological uptake.

The patient underwent radioiodine treatment with an activity of 130 mCi (patients prepared with L-T4 withdrawal), and a whole-body scan showed only minimal post-surgical remnants. Subsequent ^18^FDG/PET-CT performed after 4 months showed no residual disease (**Figure 3**). At the 1-year follow-up, the patient was free of disease: ^18^FDG/PET-CT was negative (**Figure 4**), and basal and stimulated thyroglobulin (Tg) was undetectable.

## Discussion

TKIs have revolutionized the treatment of advanced iodine-refractory thyroid cancer (1, 2, 12). Many studies have shown that these drugs can also play a role in the treatment of ATC (1, 2). Studies on treatment with multi-target TKIs, such as L, have shown conflicting results regarding the real possibility of significantly ameliorating survival. The first study was encouraging, and L was authorized in Japan for the treatment of ATC (13). However, a subsequent multicenter trial failed to show benefit, and the trial was thus stopped (NCT02657369). In another retrospective study of 23 patients, off-label use of L as a second-line treatment appeared to be more effective in mixed ATC than in pure ATC (14). This discrepancy in the data may have been dependent on the dose of the drug used (11). On the other hand, the results of treatment with D and T appear more interesting in special cases. These drugs act in a sequential manner by blocking the RAF–MEK–ERK enzymes in the MAP kinase cascade, and the clinical results of their use on *BRAF* V600E-mutated ATC have been encouraging (15–17). Treatment with D+T is now the preferred option for these patients; in fact, their use is widely discussed in the ESMO and ATA guidelines (1, 2). For ATC, there is also a strong rationale for the use of immunotherapy, especially P (18–20). The mutation burden in ATC causes an elevated immune infiltration, which the tumor can react to with the elevated expression of immune checkpoint ligands, including programmed death-ligand 1 (PD-L1). For this reason, P has been used in the treatment of ATC in combination with TKIs. L+P has especially been used in *BRAF* wild-type cancers, and a further rationale for the use of this combination is that VEGF-A can induce immunosuppression in the tumor microenvironment by inhibiting dendritic cell maturation and by reducing T-cell infiltration (21). On the other hand, D and T+P have been used successfully in *BRAF* V600E-mutated cases.

In one of the largest series (7), six patients with ATC and two patients with poorly differentiated thyroid cancer (all at stage IVC and with *BRAF* wild-type) were treated with the L+P combination after surgical intervention, followed by EBRT and systemic chemotherapy. The results were exceptionally good: the best overall response in ATC was a complete response in four out of six patients. There are also a few other reports in which P+L appeared to be effective as salvage treatment in *BRAF* wild-type cases (5, 6). In another report of *BRAF* wild-type ATC, treatment with P+L was used as an adjuvant, enabling the resection of the lesion (an apparent R0), but the patient relapsed 4 months later (8). Finally, another study showed some efficacy of treatment with P+L as the first-line therapy (after EBRT) in five patients with ATC. In a later study, the median progression-free survival (PFS) was 4.7 months (range, 0.8–5.9 months), while the median overall survival (OS) was 6.3 months. One patient was still alive at the time of data collection (12.7 months) (22).

Our case shows that treatment with L at a dose of 14 mg and P (which was used in the standard regimen) can also play a neoadjuvant role in *BRAF* V600E-mutated ATC.

This case gives rise to various speculations. First, this tumor represents a clear example of the anaplastic transformation of differentiated thyroid cancer. As demonstrated in previous studies, anaplastic transformation occurs through complex and heterogeneous mechanisms (23, 24). In this case, the molecular profiles of PTC and ATC clearly suggest that the undifferentiated tumor is derived from its differentiated counterpart. The presence of *BRAF* and *TERT* promoter mutations in both tumor components suggests that they may have a common origin and that, over time, the tumor cells clonally evolved by also acquiring the *TP53* mutation, ultimately resulting in dedifferentiation.

Second, because the P+L combination can also work in *BRAF* V600E-mutated cancers, starting with L+P is an option if time is required for molecular profiling, especially considering that the growth of ATC is extremely rapid and that the possible clinical benefit can also be quite rapid. However, as next-generation sequencing (NGS) can be time-consuming, real-time PCR can help to determine such a mutation in a shorter time. Immunohistochemistry could also be used for the diagnosis of the *BRAF* V600E mutation if other methods are not readily available.

The third issue is the dose of L used. Our previous report (11) showed a very good response with 14 mg in two cases, particularly in one case that was stage IVC and had a partial response with a PFS of 16 months. We speculated that in the complex mechanism of regulating the various MAP kinase cascades, a specific, most effective dose may exist, beyond which a hook effect occurs and the efficacy decreases. *In vitro* data appear to show that the effects of L on ATC cells are dose-dependent. However, L can act on FGR1–FGR4, PDGFR-b, RET, and c-kit, although its main effect is considered to be on VEGFR-1, VEGFR-2, and VEGFR-3 and, hence, on tumor vascularization. Other experimental models are thus needed, and studies on organoids may provide an answer to the question of the dose effect of L in *in vivo* ATC. An alternative and simpler hypothesis is that this dose represents a good middle road for these fragile patients. However, 14 mg of L does appear to be effective, with only a few adverse effects.

To the best of our knowledge, this case represents the first in which neoadjuvant treatment with L+P in *BRAF* V600E-mutated ATC has enabled complete resection of the mass and complete cure of the patient for at least 1 year.

A clinical consideration is that this result was also possible, as we only needed to obtain a small reduction in the mass, i.e., to split the tumor from the deep planes of the neck and especially from the carotid artery. This point should be kept in mind with regard to possible neoadjuvant treatments in these patients, most importantly considering that the optimal aim of surgical intervention is to perform R0 resection.

In conclusion, L and P in combination appear to be effective as a neoadjuvant treatment for ATC, as well as in *BRAF* V600E-mutated ATC. Their use could be considered when a complete resection of the cancer is possible, which should likely be done as soon as possible while awaiting the results of molecular analysis. Changing into another TKI could also be a second option if there is a specific mutation and if there is no benefit. A middle dose of 14 mg of L can be well tolerated and appears effective.

## Data availability statement

The original contributions presented in the study are included in the article/supplementary material. Further inquiries can be directed to the corresponding author.

## Ethics statement

Written informed consent was obtained from the individual(s) for the publication of any potentially identifiable images or data included in this article.

## Author contributions

DB: Writing – original draft, Writing – review & editing. RF: Writing – original draft. MP: Writing – original draft. PL: Writing – original draft. CG: Writing – original draft. LT: Writing – original draft. EM: Investigation, Writing – original draft. GM: Writing – original draft.
